# Effects of Weizhuan’an on rats with precancerous lesions of gastric cancer based on regulating gastric mucosal microflora and inflammatory factors

**DOI:** 10.3389/fphar.2024.1446244

**Published:** 2024-08-16

**Authors:** Yuting Lu, Huayi Liu, Jiaju Shang, Yijia Mao, Lingkai Meng, Changbai Gao

**Affiliations:** ^1^ Guangdong Second Provincial General Hospital, Integrated Chinese and Western Medicine Postdoctoral Research Station, School of Medicine, Jinan University, Guangzhou, Guangdong, China; ^2^ Graduate School, Tianjin University of Traditional Chinese Medicine, Tianjin, China; ^3^ Department of Digestion, Tianjin Academy of Traditional Chinese Medicine Affiliated Hospital, Tianjin, China; ^4^ Department of Nephropathy, Second Affiliated Hospital of Tianjin University of Traditional Chinese Medicine, Tianjin, China

**Keywords:** precancerous lesion of gastric cancer, gastric mucosa, traditional Chinese medicine, gastric mucosal microflora, inflammatory factors, Weizhuan’an prescription

## Abstract

**Objectives:**

This study aimed to observe the intervention of Weizhuan’an prescription on rats with precancerous lesions of gastric cancer (PLGC) as well as its regulation on gastric mucosal microflora and inflammatory factors and explore the pharmacodynamic mechanisms of Weizhuan’an Formula.

**Methods:**

The rats were classified into the blank control group (BCG); low-, medium-, and high-dose groups of Weizhuan’an prescription (LDG, MDG, and HDG, respectively); and natural recovery group (NRG) at random. The rats in the traditional Chinese medicine (TCM) group were given corresponding doses of Weizhuan’an formula, while the rats in the NRG and BCG were given an equivalent volume of distilled water for 12 weeks. After that, gastric mucosa samples of rats were collected to observe the general and pathological changes in the gastric mucosa; the changes in gastric mucosal microflora were detected by 16S rDNA amplicon sequencing, and the inflammatory factors were analyzed by cytokine antibody microarray and Western blotting.

**Results:**

The results suggest that compared with the BCG, the pathology of gastric mucosa and gastric mucosal microflora and inflammatory factors in rats with PLGC have changed significantly, while Weizhuan’an formula effectively improved them, especially in the MDG and HDG (*p* < 0.05). Compared with the NRG, the abundance of probiotics such as *Lactobacillus* and *Veillonella* were increased, while the abundance of pathogens such as *Proteobacteria* and *Pseudomonas* was decreased (*p* < 0.05, *p* < 0.01), and the relative contents of IL-2, IL-4, IL-13, and MCP-1 in gastric mucosa were decreased (*p* < 0.05). Moreover, it can upregulate the DNA-binding transcriptional regulator, ABC type multidrug transport system, and related enzymes and affect the signaling pathways such as viral protein interaction with cytokine and cytokine receptor and T cell receptor signaling pathway significantly (*p* < 0.05, *p* < 0.01), which can promote drug absorption and utilization and repair damaged gastric mucosa.

**Conclusion:**

The study confirmed that Weizhuan’an prescription can treat rats with PLGC by regulating gastric mucosal microflora and inflammatory factors.

## 1 Introduction

Gastric cancer (GC) is one of the commonest gastrointestinal malignant tumors worldwide, which seriously threatens human health owing to its rising morbidity along with high mortality (Fitzmaurice et al., 2017; Zheng et al., 2022). It is difficult to apply primary prevention due to the complicated causes of GC, and therefore the intervention on precancerous lesions of gastric cancer (PLGC) has always been a hot spot in clinical research. According to the Chinese Integrated Clinical Management Guidelines for Precancerous Lesions of Gastric Cancer (Wang et al., 2022), common therapies for PLGC include eradicating Hp (positive cases) and short-term use of acid suppressants, mucosal protectors, etc., as well as using endoscopic surgical techniques combined with botanical drugs to treat high-grade dysplasia and early GC. Folic acid, antioxidant vitamins, etc. can also reduce the risk of GC to a certain extent. Long-term use of Western medicine is usually avoided in clinical practice due to the common side effects of using them, such as abdominal pain, nausea, and gastrointestinal bloating. As the treatment cycle for PLGC is relatively long, botanical drugs are widely used due to its advantages of good efficacy and minimal side effects. The combination of traditional Chinese medicine and Western medicine has significant advantages in treating PLGC. There is a complex gastrointestinal microecosystem in the body, and there are various species of gastric microflora, although they are fewer than intestinal microflora species (Nardone and Compare, 2015; Sung et al., 2016). Studies have confirmed that the structure and abundance of gastric microflora in patients with GC and PLGC are significantly different from that in the healthy controls. The imbalance of gastric microflora can induce gastric diseases such as gastritis and gastric ulcer, which is tightly related to the occurrence of GC (Sohn et al., 2017; Yu et al., 2017). Moreover, the relationship between the gastric inflammatory environment and GC and PLGC is another research highlight at present (Gullo et al., 2019; Zhou and Yang, 2019). A normal immune response can repair damaged tissues and maintain homeostasis. However, when the body is in a state of repeated infection or injury, inflammatory stimulation will inhibit the repair of damaged gastric mucosa (Sounni and Noel, 2013), eventually leading to canceration of gastric mucosal epithelial cells (Schulz et al., 2019). Some studies (Khatoon et al., 2018; Sultana et al., 2018) have suggested that although the imbalance of intragastric microflora may mediate inflammation to damage the gastric mucosa, the final clinical outcome of patients depends on the body’s inflammatory response. Conversely, the strength of the inflammatory response is also influenced by the intragastric microenvironment (Bockerstett and DiPaolo, 2017; Chen et al., 2018). Existing studies have confirmed that traditional Chinese medicine (TCM) has obvious effects in inhibiting gastric pathogenic bacteria (Lian et al., 2017; Deng, 2020), regulating inflammatory factors, and repairing gastric mucosal damage (Li et al., 2015; Yu et al., 2018). For the most part, there is still a lack of experimental research on the regulation of gastric microecological immunity by TCM for treating PLGC. Weizhuan’an prescription, created by the late Professor Gao Jinliang, a famous gastroenterologist, is an empirical prescription for the treatment of PLGC. Our previous research has confirmed that Weizhuan’an prescription can effectively relieve the clinical symptoms of patients and improve the pathology of the gastric mucosa (Wang et al., 2017; Meng et al., 2020). In order to clarify its mechanism, this experiment from the perspective of regulating gastric mucosal microflora and inflammatory factors provides a view to opening up a new direction for clinical application.

## 2 Experimental materials

### 2.1 Experimental animals

Hundred male and healthy SPF Wistar rats, aged 6 weeks, weighing 175 ± 25 g, were provided by SPF (Beijing) BIOTECHNOLOGY Co., Ltd., the animal license No. is SCXK (Beijing) 2019-0010. It was raised in the SPF Laboratory room of the Animal Experiment Center of Tianjin University of Traditional Chinese Medicine (TUTCM), room No. 337. The room temperature was 23 ± 2°C, and the relative humidity was 55 ± 5%, with a 12-h light and 12-h dark cycle. This experiment complies with the regulations of the Ethics Committee of the Animal Experiment Center of TUTCM, batch No. TCM-LAEC2020076.

### 2.2 Experimental medicine

The composition of Weizhuan’an prescription is as follows: 15 g Radix pseudostellariae (batch No. 21010101), 15 g *Poria cocos* (batch No. 194200901), 10 g fried *Atractylodes macrocephala* Koidz (batch No. 200911006), 30 g *Astragalus mongholicus* (batch No. 2103260021), 10 g *Curcuma zedoaria* (batch No. 2104050162), 30 g Hedyotis diffusa Willd (batch No. 201211002), 30 g *Polygonum cuspidatum* (batch No. 255190101), and 3 g pseudo-ginseng (batch No. 901180602). Chinese medicinal materials were purchased from Anhui Xiehecheng Co., Ltd., Beijing Hongji Pharmaceutical Co., Ltd., Beijing Qiancao Herbal Pieces Co., Ltd., and Bozhou Huqiao Pharmaceutical Co., Ltd. The concentrated liquid of botanical drugs was decocted by the national standard preparation and provided by the pharmacy of Tianjin Academy of Traditional Chinese Medicine Affiliated Hospital. The specific method for decoction is as follows: soak the dried medicinal botanical drugs (except pseudo-ginseng) and eight times of cold distilled water in the casserole for 30 min. Boil it over high heat and then turn to low heat and decoct for 30 min. Then, filter the decoction to remove the drugs and concentrate the filtered liquid to 200 mL. Store in the refrigerator of the Animal Experiment Center of TUTCM at 4°C away in dark conditions. Pseudo-ginseng was stored in powder form at room temperature and away from light. The main extract of Weizhuan’an prescription was quantitatively analyzed by high-performance liquid chromatography (HPLC) and liquid chromatography-tandem mass spectrometry (LC-MS/MS). The results showed the presence of notoginsenoside R1 (27.0 mg·L^−1^), ginsenoside Rb1 (87.2 mg·L^−1^), ginsenoside Rg1 (111 mg·L^−1^), kaempferol (29.6 mg·L^−1^), quercetin (1.99 mg·L^−1^), astragaloside IV (20.1 mg·L^−1^), polydatin (95.3 mg·L^−1^), heterophyllin (24.7 mg·L^−1^), atractylenolide Ⅱ (1.21 mg·L^−1^), atractylenolide III (10.0 mg·L^−1^), and poricoic acid A (0.101 mg·L^−1^).

95% N-methyl-N′-nitro-N-nitrosoguanidine (MNNG), provided by the Tokyo Chemical Industry Development Co., Ltd., specification: 25 g*1 bottle, batch No. P1734775, was stored in the refrigerator of the Animal Experiment Center of TUTCM at 4°C away from light. The SPF compound diet for rats was formulated by Beijing HFK Bioscience Co., Ltd., in the proportion of 99.77% ordinary diet and 0.2% sodium chloride and 0.03% ranitidine, production license No. Beijing (2019) 06076. Purified sodium chloride was provided by Tianjin Zhiyuan Chemical Reagent Co., Ltd., specification: 500 g*1 bottle. Ranitidine was provided by Jiangxi Huiren Pharmaceutical Co., Ltd., specification: 0.15 g*30 tablets*1 bottle, batch No. H44021173. SPF ordinary diet for rats was provided by the Animal Experiment Center of TUTCM, and the basic ingredients are corn, flour, wheat bran, soybean meal, imported fish meal, oil, stone powder, calcium hydrogen phosphate, salt, complex microorganisms, and trace elements.

### 2.3 Experimental apparatus

CX31 microscope and graphic analysis system (Olympus, Japan); SH-118 electronic scale (Shengheng, China); RM2125 pathological microtome (Leica, Germany); 7FB Water Bath-Slide Drier (Xiaogan Yaguang, China); HP-D Sheet dryer (Tianjin Tianli Aviation, China); JBM-B Embedding machine (Tianjin Jiusheng, China); 101-1AB Electric blast drying oven (Tianjin Taisote, China); ASP200S totally enclosed vacuum biological tissue dehydrator (Leica, Germany); NovaSeq PE250 sequencer (Illumina, United States); rat inflammation array Q1 (QAR-INF-1) kit (RayBiotech, United States); InnoScan 300 Microarray scanner (Innopsys, France); Wellwash Versa Microplate Washer (Thermo Scientific, United States); RT-6100 Microplate Reader (Rayto, China); F6/10 Micropipette Grinder (Jingxin technology JX-FSTGRP, China); TGL-16gR Table-type High-speed Refrigerated Centrifuge (Shanghai Anting, China); DL-40B Desktop low-speed centrifuge (Shanghai Anting, China); Mini-4s Multifunctional centrifuge (Labfish, China); VM-300 Multifunctional vortex mixer (Labfish, China); 180-1600 Casting Stand (Shanghai Tanon, China); SK-O180-E Decolorization Shaker (DragonLAB, China); DYY-7C Transfer electrophoresis apparatus (Beijing Liuyi, China); VE180 Electrophoresis equipment Tanon, China); VE186 Transmembrane equipment (Shanghai Tanon, China); XB-50 Ice machine (Scientz, China); SCIENTZ-1500F Ultrasonic Processor (Scientz, China); 5200 Chemiluminescence Apparatus (Shanghai Tanon, China).

### 2.4 Experimental reagents

P0013B RIPA lysate (Beyotime Biotechnology, China); P1010 protease inhibitor (Beyotime Biotechnology, China); ST506 PMSF (100 mM) (Beyotime Biotechnology, China); P1081 phosphatase inhibitors (Beyotime Biotechnology, China); P0012 BCA protein quantitative detection kit (Beyotime Biotechnology, China); P0015 SDS-PAGE SDS-PAGE loading buffer (5X) (Beyotime Biotechnology, China); P0012A SDS-PAGE gel preparation kit (Beyotime Biotechnology, China); 26617 protein marker (Thermo, United States); ISEQ10100 PVDF membrane 0.22 μm (Millipore, United States); P0216 defatted milk powder (Beyotime Biotechnology, China); ST825 Tween-20 (Beyotime Biotechnology, China); P0018AS ECL chemiluminescent solution (Beyotime Biotechnology, China); 66009-1 β-actin (43KD) (Proteintech, United States); PB0059 IL-2 (18KD) (Boster, China); BA0980 IL-4 (17KD) (Boster, China); A00077-2 IL-13 (19KD) (Boster, China); BA 1843-2 MCP-1 (25KD) (Boster, China); SA00001-2 HRP-labeled goat anti-rabbit (Proteintech); SA00001-1 HRP-labeled goat anti-mouse (Proteintech); P0021A transfer buffer (Beyotime Biotechnology, China); P0014 electrophoretic buffer (Beyotime Biotechnology, China); ST673 TBS-T buffer (Beyotime Biotechnology, China); P0023 WB antibody diluent (Beyotime Biotechnology, China).

## 3 Experimental methods

### 3.1 Modeling method

After 1 week of adaptive feeding, the rats were randomly divided into two groups with a random number table: 14 in the blank control group (BCG) and 86 in the model control group (MCG). The MCG was established by compound methods. In the 1st–26th week, MNNG and distilled water were prepared into a 1 g·L^−1^ stock solution and stored in a refrigerator at 4°C away from light every week. Then, the 100 μg·mL^−1^ MNNG solution was diluted with clean drinking water and filled into black bottles and supplied *ad libitum* to rats every day. The SPF compound diet was also supplied ad libitum to rats. From the 27th–30th week, it was prepared into 20 μg·mL^−1^ MNNG solution (the co-solvent shall be added in a ratio of 1 g MNNG to 10 mL dimethyl sulfoxide) and stored in refrigerator at 4°C away from light every week and then administered to the rats with the dose of 5 mL·kg^−1^ through gastric tubes every day. The SPF ordinary diet was administered to rats in the ratio of eating for 1 day and fasting for 1.5 days. In addition, the BCG had *ad libitum* access to SPF ordinary diet and drinking water.

### 3.2 Determination of the model of PLGC

The pathological diagnosis standard referred to the *Chinese consensus on chronic gastritis* (Chinese Society of Gastroenterology (CSGE), 2017). Moderate dysplasia and atrophy or intestinal metaplasia of gastric mucosa can confirm the success of modeling. From the 24th weekend, two rats from the MCG were randomly selected and killed based on the random number table fortnightly. Two tissues of gastric antral mucosa were taken and fixed with 4% paraformaldehyde, made into paraffin sections, and stained with hematoxylin and eosin (HE), and then the pathological changes were observed by using a light microscope. At the 30th weekend, the pathological section of the gastric mucosa in one of the selected rats showed inflammatory cell infiltration, moderate atrophy, and dysplasia, and the other showed mild intestinal metaplasia and moderate dysplasia, indicating that the model of PLGC was successfully established.

### 3.3 Drug intervention

After successful modeling, 76 rats in the MCG were divided into the low-dose group (LDG), middle-dose group (MDG), high-dose group (HDG), and natural recovery group (NRG) by random number table, with 19 rats in each group. Referring to the formula [the dose of the crude drug of rats (g·kg^−1^) = 143 g·d^−1^ of adult clinical crude drug dose/70 kg of adult weight × 6.25], we calculated the daily dose of the equivalent dosage of crude drug as 12.76 g·kg^−1^ (the extract is 7.284 mg·kg^−1^). The drug concentration gradient to the dose of the extract of the LDG, MDG, and HDG is 3.642, 7.284, and 14.570 mg·kg^−1^, respectively. In the 31st–42nd weeks, the rats in the LDG, MDG, and HDG were given a corresponding dose of Weizhuan’an (7 mL kg^−1^) at 7:30–8:30 every morning, and the rats in the BCG and NRG were given an equal dose of distilled water.

### 3.4 Observation index

We draw up the diagnostic and scoring criteria for PLGC, referring to the pathological diagnosis and grading standard of *Chinese consensus on chronic gastritis* (CSGE, 2017) and *Endoscopic classification and grading criteria for chronic gastritis* (Association of Digestive Endoscopy, Chinese Medical Association (CMA), 2004). The quantified score: normal = 0; mild = 1; moderate = 2; severe = 3; early gastric cancer (EGC) = 4; advanced gastric cancer (AGC) = 5.

### 3.5 Sampling methods

All rats were starved for 36 h without water after the intervention, and then they were anesthetized by intraperitoneal injection of 0.3% pentobarbital sodium at 10 mL·kg^−1^. After that, the abdomen was quickly opened with sterile surgical scissors, done on a sterile operating table, and the whole stomach was taken out. After gentle washing with pH 6.8 phosphate buffer, they were cut along the greater curvature of the stomach, and four pieces of the gastric antrum mucosa were taken and rinsed in phosphate buffer. One piece of the gastric mucosa was stored in a 2-mL Eppendorf (EP) tube with 4% paraformaldehyde, away from light. Ten samples were taken from each group with a random number table, made into paraffin sections, stained with HE, and observed by a light microscope. Three pieces of gastric mucosa were added into 2-mL cryogenic vials, quickly frozen in liquid nitrogen, and stored at −80°C. Subsequently, 16S rDNA amplicon sequencing, cytokine antibody microarray, and Western blotting were performed following the standard method. All rats were killed by cervical dislocation after sampling.

### 3.6 16S rDNA amplicon sequencing operation procedure

Ten samples were taken from each group with a random number table. Subsequently, the deoxyribonucleic acid (DNA) of samples was extracted by cetyltrimethylammonium bromide (CTAB) buffer, and then polymerase chain reaction (PCR) amplification and purification were carried out. The library was constructed by using the NEBNext^®^ Ultra™ IIDNA Library Prep Kit, and the qualification of the quality control of the library was confirmed by the quantitative detection with Qubit and quantitative real-time PCR (Q-PCR). The raw data were obtained by sequencing with NovaSeq PE250, and the clean read data were obtained by splicing and filtering, and then the final amplicon sequence variants (ASVs) were obtained by DADA2 denoising based on clean read data.

### 3.7 Cytokine antibody microarray procedure

The expression of inflammatory factors in gastric mucosa in each group was detected by the QAR-INF-1 kit. Ten gastric mucosa samples were randomly taken from each group. After all samples were lysed, the protein concentration of the cell lysate was detected by the BCA protein assay kit, and sampled 100 μL at 500 μg·mL^−1^. The glass slide was completely air-dried, and cytokine standard dilutions were prepared. The cytokine antibody microarray procedure was performed according to the manufacturer’s manual: 1) add 100 µL sample diluent into each well, incubate for 1 h at room temperature, and block the slides. 2) Decant the buffer solution from the well, add 100 µL standard cytokines and sample into each well, and incubate overnight in a shaker at 4°C. 3) Wash the slide by using a microplate washer (Thermo Scientific Wellwash Versa). First, select 1X Wash Buffer I to wash the slide, each well with 250 µL buffer ten times, and each high-intensity vibration lasts for 10 s. Dilute 20X Wash Buffer I with deionized water. Then, select the 1X Wash Buffer II for washing, each well with 250 µL buffer six times, and each high-intensity vibration lasts for 10 s. Dilute 20X Wash Buffer II with deionized water. 4) Incubate with biotinylated antibody cocktail and wash. 5) Incubate with Cy3 equivalent dye-streptavidin and wash. 6) Fluorescence detection. Scan parameters: wavelength: 532 nm; resolution: 10 µm. 7) Adopt QAR-INF-1 data analysis software for data analysis.

### 3.8 Western blotting procedure

Three gastric mucosa samples were randomly selected from each group. The total protein of the gastric mucosa was extracted, and the concentration was detected by the BCA protein assay kit. Adjust all protein samples to a uniform concentration of 3 μg·μL^−1^. Add 5X reduced protein loading buffer into the protein solution at the ratio of 4:1, and transfer to a boiling water bath for 5 min. Then, SDS-PAGE electrophoresis was carried out, the voltage of the concentrated gel was 70 V, and the voltage of the separated gel was 100 V at constant pressure. Add enough electrophoresis solution to the electrophoresis tank and load the sample for electrophoresis. Then, the protein was transferred to a membrane and placed for 60 min under a constant current of 200 mA, and the reaction was completed with primary and secondary antibodies, chemiluminescence, and automatic imaging. ImageJ software was used to analyze the gray value of the protein bands.

### 3.9 Statistical methods

The pathological analysis and Western blotting results of the gastric mucosa in rats were analyzed by Statistical Product Service Solutions (SPSS) 25.0. The measurement data of normal distribution were represented by mean standard deviation (‾*x* ± *s*), the paired *t* test was used in intra-group comparison, and the one-way ANOVA was used in inter-group comparison. The measurement data of non-normal distribution were expressed by interquartile range (IQR) *M* (*P25*; *P75*). The rank sum test was adopted for intra-group comparison, and the Kruskal–Wallis test was adopted for inter-group comparison. *p* < 0.05 indicated statistical differences.

The ASVs obtained by 16S rDNA amplicon sequencing were processed in two ways. First, by annotating the species of ASVs and then performing alpha diversity analysis, including alpha diversity index and inter-group difference analysis. Second, multi-sequence alignment was used for ASVs, and the phylogenetic tree was constructed. It was visually displayed by principal co-ordinates analysis (PCoA) and non-metric multidimensional scaling (NMDS), and the differentially abundant features of microflora were analyzed by Mothur metastats command (MetaStats) and linear discriminant analysis effect size (LEfSe). In addition, the correlation of microflora was calculated by *Spearman* correlation analysis, and the functional abundance of microflora was predicted by PICRUSt2 based on the clusters of orthologous groups (COG).

After the normalization of raw data obtained by cytokine antibody microarray by RayBiotech software, the resulting data are selected for analysis. The analysis method is moderated t-statistics, and the data package used is limma, from R/Bioconductor. Differential proteins were screened by *p-value* and logFC. The parameter threshold is that the fold change >1.2 or <0.83, that is, the absolute value of logFC >0.263, and *p* < 0.05. The Kyoto Encyclopedia of Genes and Genomes (KEGG) enrichment analysis was adopted to predict the functions of differential inflammatory factors.

## 4 Results

### 4.1 Pathology of the gastric mucosa in rats

**FIGURE 1:**
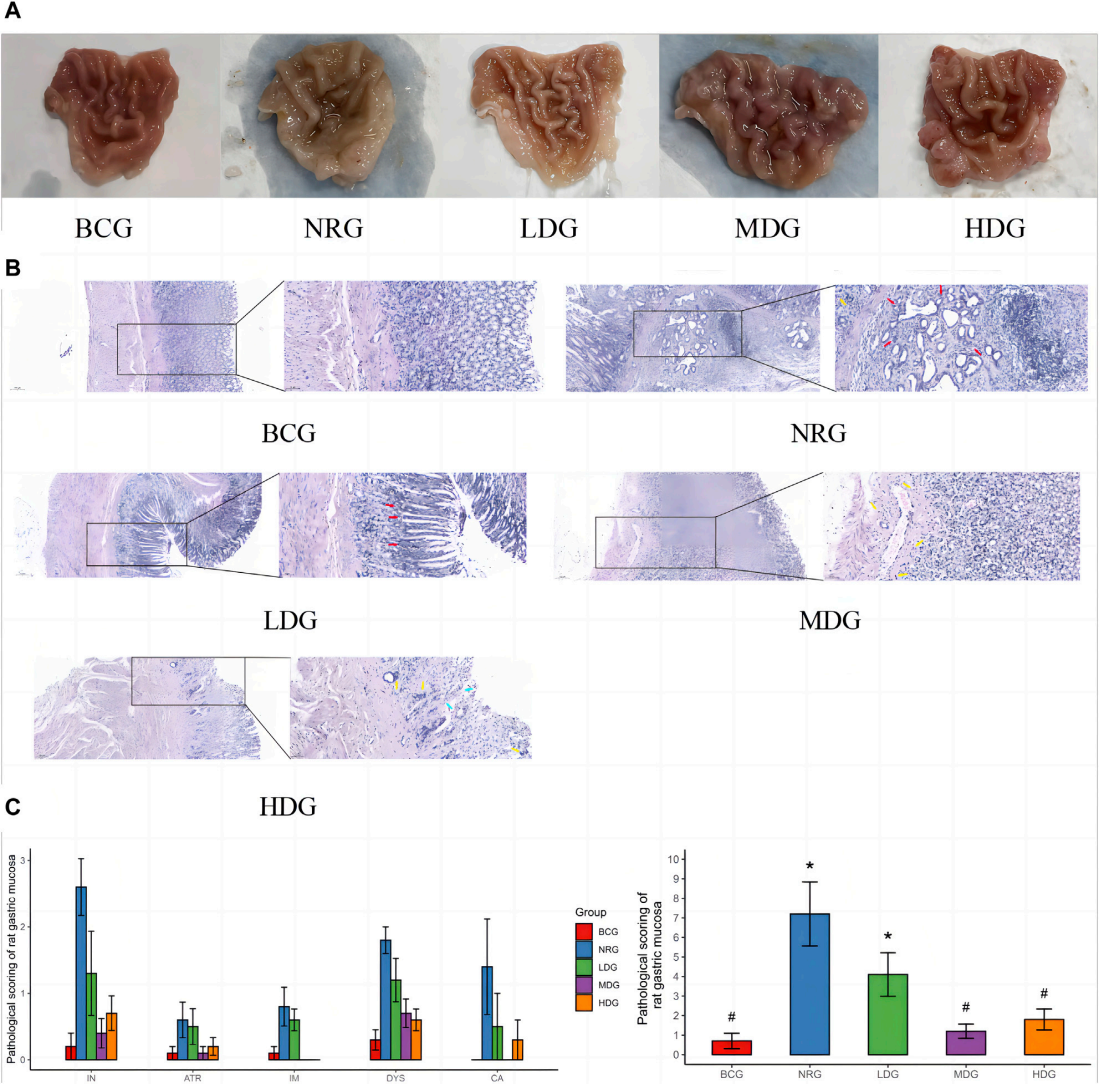
**(A)** General conditions of the gastric mucosa of rats in each group at the 42nd weekend. **(B)** HE staining sections of the gastric mucosa of rats in each group at the 42nd weekend (×100/×400) Note: dysplasia (red arrow); atrophy or intestinal metaplasia (blue arrow); and inflammation (yellow arrow). **(C)** Pathological score of the gastric mucosa of rats in each group at the 42nd weekend Note: * indicates compared with the BCG, *p* < 0.05; # indicates compared with the NRG, *p* < 0.05.

The general conditions of the gastric mucosa of rats in each group can be observed in Figure 1A. It was found that the general condition of gastric mucosa in the BCG was the best, while that in the LDG, MDG, and HDG was improved to varying degrees, which was better than that in the NRG. The HE staining sections of rat gastric mucosa at the 42nd weekend are shown in Figure 1B. There was a significant difference in the pathological changes of the gastric mucosa among groups. In the BCG, the gastric mucosa of rats was in good condition, the morphology of gastric mucosa cells was regular, the number of intrinsic glands did not decrease, intestinal metaplasia and dysplasia did not exist, and there was no infiltration of monocytes, neutrophils, or cancer cells in the mucosa. In the NRG, the overall condition of the gastric mucosa was poor, the number of intrinsic glands in the gastric mucosa was reduced, there was higher infiltration of monocytes and neutrophils into gastric lamina propria, and the intestinal metaplasia area in some rats exceeded the total area of the gastric mucosa by one-third. It can be seen in Figure 1C that the pathological score of the BCG is the lowest while the NRG is the highest, that of the Weizhuan’an group was intermediate between the two groups, and that of the MDG and HDG was obviously improved. Specifically, there was no statistical difference in atrophy or canceration of rats in each group. Compared with the NRG, there were significant differences in the BCG, MDG, and HDG in terms of chronic inflammation, intestinal metaplasia, and dysplasia (*p* < 0.05). There were significant differences in each group compared with the NRG in terms of active inflammation (*p* < 0.05).

### 4.2 Relative abundance and abundance clustering of gastric mucosal microflora in rats

**FIGURE 2:**
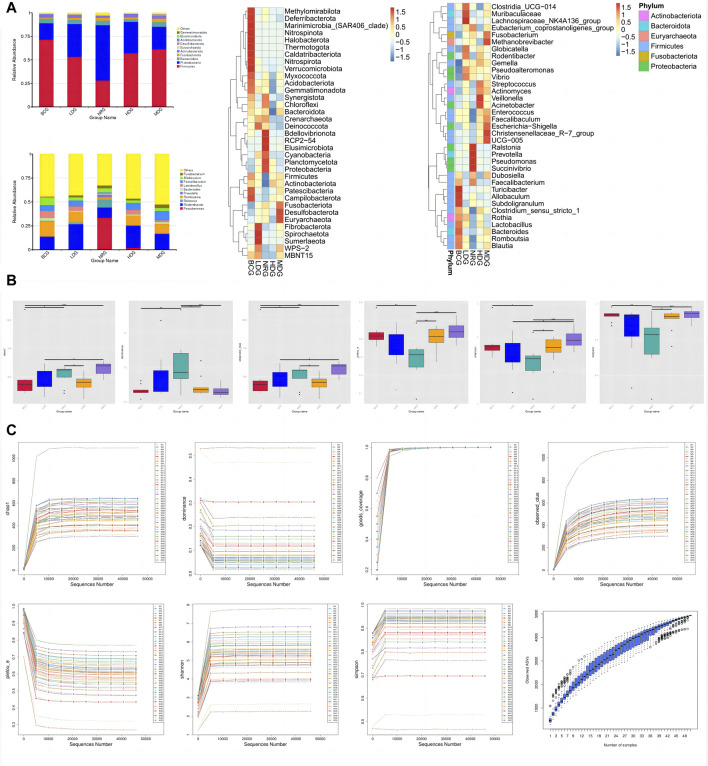
**(A)** Histograms and heat maps of the relative abundance of gastric mucosal microflora (phylum and genus levels). **(B)** Boxplots of alpha diversity indices of gastric mucosal microflora of rats in each group. **(C)** Rarefaction curves and species accumulation boxplots of alpha diversity indices.

The histograms (top 10) and heat maps (top 35) of the relative abundance of gastric mucosal microflora of rats in phylum and genus levels were drawn with QIIME 2 based on the annotation of the microflora abundance table, which can be referred in Figure 2A. There were 46 bacterial phyla and 896 bacterial genera with significant differences between groups. The relative abundance of five phyla in the top 10 was more than 1%, *Firmicutes*, 54.45%; *Proteobacteria*, 33.63%; *Bacteroidota*, 4.34%; *Actinobacteriota*, 3.39%; and *Fusobacteriota*, 2.32%. The relative abundance of all genera in the top 10 was more than 1%, except for *Prevotella*.

### 4.3 Alpha diversity analysis

The diversity of microflora was evaluated by alpha diversity analysis. As shown in Figure 2B, compared with the species diversity of the NRG, there were significant differences in the BCG, MDG, and HDG (*p* < 0.001), among which the MDG was the most significant. The rarefaction curves of alpha diversity indices indicate that the average effective sequence is 6.9 w/sample. The rarefaction curves tend to be flat when the sequence is about 4.6 w (cutoff = 45945). In addition, the species accumulation boxplots of alpha diversity indices tend to be flat gradually when the number of samples is approximately 45, which shows that the sampling is reasonable and sufficient, and it is suitable for subsequent analysis. See Figure 2C for details.

### 4.4 Beta diversity analysis

**FIGURE 3:**
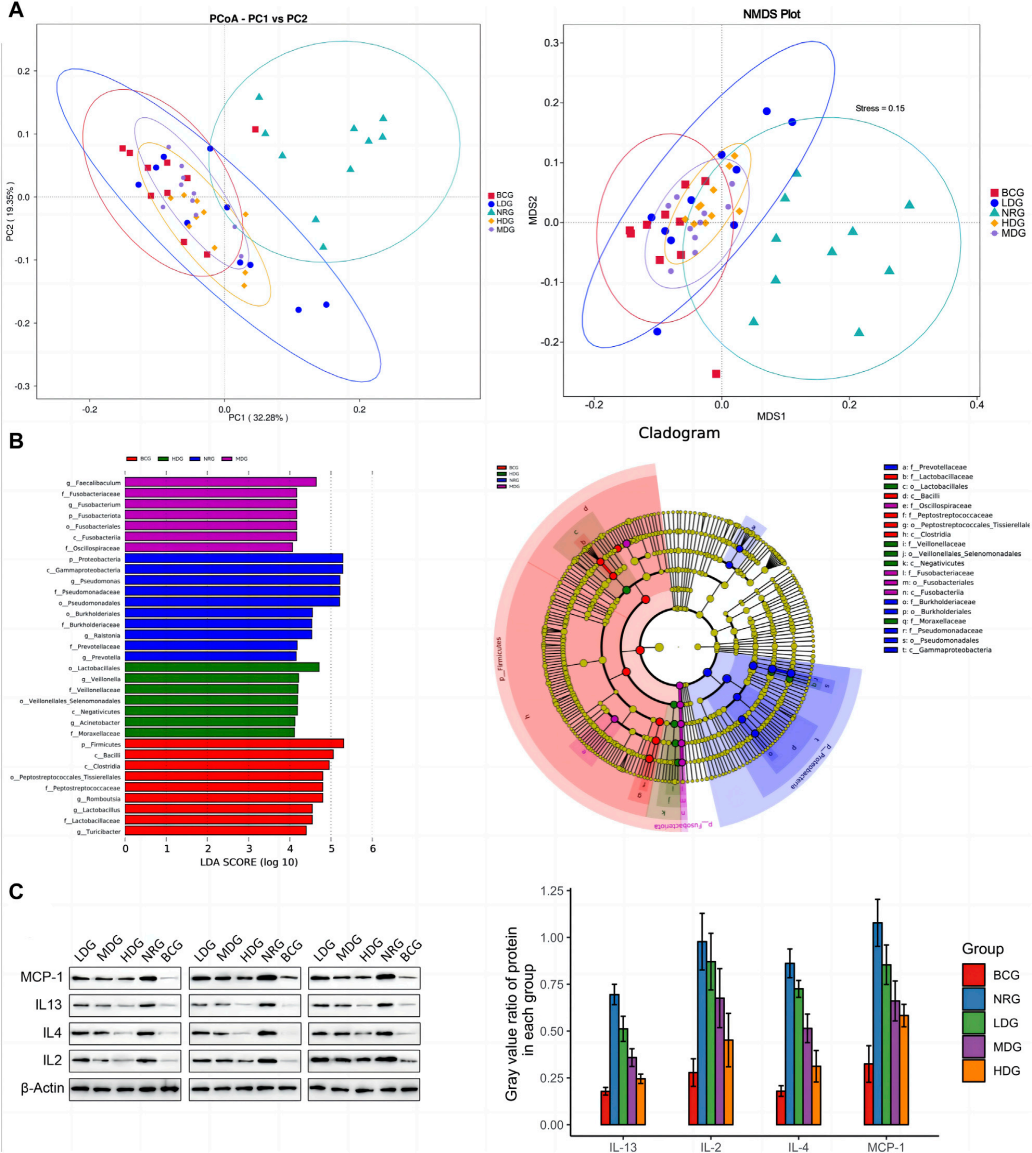
**(A)** PCoA and NMDS analysis of duodenal mucosal microflora of rats in each group. **(B)** LEfSe analysis of duodenal mucosal microflora of rats in each group. **(C)** Gray value and gray value ratio of each protein in the gastric mucosa of rats.

It can be seen in Figure 3A that the first principal component (PC1) and the second (PC2) of the PCoA with weighted UniFrac are 32.28% and 19.35%, respectively. The stress value of NMDS with weighted UniFrac is 0.15, which is lower than the threshold value of 0.2. All of them form five clusters with unequal overlapping parts, and the NRG is quite different from other groups. Combined with ANOSIM, it can be seen that R > 0 in each group, indicating that the difference between groups is greater than that within groups, and the difference is significant (*p* < 0.05) among groups, except for the LDG and NRG, which indicates that the conclusion is reliable.

### 4.5 Analysis of differential abundance

The results of MetaStats analysis suggest that compared with the gastric mucosal microflora of the NRG, there were significant differences in the BCG, MDG, and HDG (*p* < 0.05, *p* < 0.01). Refer to Table 1, 2 for phylum and genus levels with the top six in relative abundance and with significant differences between groups. There were 33 biomarkers combined with LEfSe analysis, as shown in Figure 3B.

**TABLE 1:** Gastric mucosal microflora of rats with remarkable differences between groups in phylum level.

BCG vs NRG		MDG vs NRG		HDG vs NRG	
Species	Discrepancy	Species	Discrepancy	Species	Discrepancy
*Firmicutes*	↑	*Bacteroidota*	↑	*Fusobacteriota*	↓
*Proteobacteria*	↓	*Euryarchaeota*	↓	*Euryarchaeota*	↓
*Actinobacteriota*	↑	*Actinobacteriota*	↓	*Proteobacteria*	↓
*Elusimicrobiota*	↓	*Acidobacteriota*	↓	*Desulfobacterota*	↓
*Patescibacteria*	↑	*Chloroflexi*	↓	*Acidobacteriota*	↓
*Verrucomicrobiota*	↑	*Elusimicrobiota*	↓	*Gemmatimonadota*	↓

**TABLE 2:** Gastric mucosal microflora of rats with remarkable differences between groups in genus level.

BCG vs NRG		MDG vs NRG		HDG vs NRG	
Species	Discrepancy	Species	Discrepancy	Species	Discrepancy
*Pseudomonas*	↓	*Pseudomonas*	↓	*Lactobacillus*	↑
*Ralstonia*	↓	*Ralstonia*	↓	*Fusobacterium*	↓
*Romboutsia*	↑	*Romboutsia*	↑	*Pseudomonas*	↓
*Lactobacillus*	↑	*Prevotella*	↓	*Acinetobacter*	↑
*Faecalibaculum*	↑	*Bacteroides*	↑	*Veillonella*	↑
*Turicibacter*	↑	*Lactobacillus*	↑	*Lachnospiraceae*	↓

Note: ↑indicates that the relative abundance of group 1 is higher than that of group 2, ↓ indicates the opposite.

### 4.6 Data statistics of cytokine antibody microarray

The average value of all sample data is normalized, and the concentration values of inflammatory factors in the samples are calculated based on the standard cytokine data; see Table 3 for specific data.

**TABLE 3:** Data statistics of the gastric mucosal inflammatory factors of rats.

Target	LOD (pg/mL)	% below LOD	% above LOD but <3×LOD	% in best confidence	% above maximum
IFN-γ	0.2	56.0	20.0	24.0	0.0
IL-1α	1.2	82.0	4.0	14.0	0.0
IL-1β	50.9	8.0	30.0	62.0	0.0
IL-2	3.9	28.0	56.0	16.0	0.0
IL-4	0.2	28.0	40.0	32.0	0.0
IL-6	5.2	42.0	0.0	58.0	0.0
IL-10	4.3	2.0	4.0	94.0	0.0
IL-13	2.7	38.0	26.0	36.0	0.0
MCP-1	1.6	0.0	0.0	100.0	0.0
TNF-α	443.0	2.0	6.0	92.0	0.0

Note: the blue font represents the data with a high proportion; italics represent relatively low data; limit of detection (LOD) represents the lowest concentration value that can be detected in the confidence interval. Unit: pg·mL^−1^.

### 4.7 Differential inflammatory factors of rat gastric mucosa

The average expression and difference of inflammatory factors in the gastric mucosa of rats in each group are given in Table 4. The results showed that eight differential inflammatory factors were detected. The BCG had the largest difference compared with the other groups, and the relative content of inflammatory factors was significantly decreased (*p* < 0.05), except that IL-13 was increased compared with the LDG. The second is the difference between the NRG and other groups. Compared with the NRG, the levels of IL-2, IL-4, and IL-13 in the LDG were significantly reduced (*p* < 0.05); the levels of IL-4 and IL-13 in the MDG were significantly reduced (*p* < 0.05); and the levels of IL-2, IL-4, and MCP-1 in the HDG were significantly reduced (*p* < 0.05).

**TABLE 4:** Average expression of inflammatory factors in the gastric mucosa of rats in each group.

Group	IL-2	IL-4	IL-10	MCP-1	IFN-γ	TNF-α	IL-13	IL-1β
BCG	1.600^#^	0.051^#^	4.321^#^	4.359^#^	0.000^#^	10.365^#^	2.612	6.237^#^
NRG	3.784*	0.890*	5.778*	5.217*	0.595*	11.663*	3.086	7.581*
LDG	2.501^#^	0.476*^#^	5.321*	5.194*	0.559*	11.063*	1.013*^#^	6.937*
MDG	3.218*	0.572*^#^	5.213*	5.088*	0.293	11.219	1.742^#^	7.751*
HDG	2.565^#^	0.527*^#^	5.254*	4.570^#^	0.311	11.255*	2.229	7.501*

Note: * indicates compared with the BCG, *p* < 0.05; # indicates compared with the NRG, *p* < 0.05.

### 4.8 Western blotting analysis

Western blotting (WB) was adopted to verify the results obtained by the cytokine antibody microarray. The results showed that the expression levels of all inflammatory factors were the highest in the NRG and the lowest in the BCG, which was consistent with the results of microarray analysis. See Figure 3C for the gray value and gray value ratio of each protein in the gastric mucosa of rats. The results showed that compared with the BCG, there are significant differences in IL-2, IL-4, IL-13, and MCP-1 in the NRG, LDG, and MDG (*p* < 0.05). Compared with the NRG, the levels of IL-4, IL-13, and MCP-1 in the MDG were significantly reduced (*p* < 0.05). The levels of IL-2, IL-4, IL-13, and MCP-1 in the HDG were significantly reduced (*p* < 0.05). In general, the overall trend of the difference between the results of WB and cytokine antibody microarray is consistent, while there are certain differences in significance, which may be caused by the bias caused by the difference in detection methods and sample size.

### 4.9 Correlation analysis of gastric mucosal microflora of rats

#### 4.9.1 Analysis of the interaction among gastric mucosal microflora of rats

**FIGURE 4:**
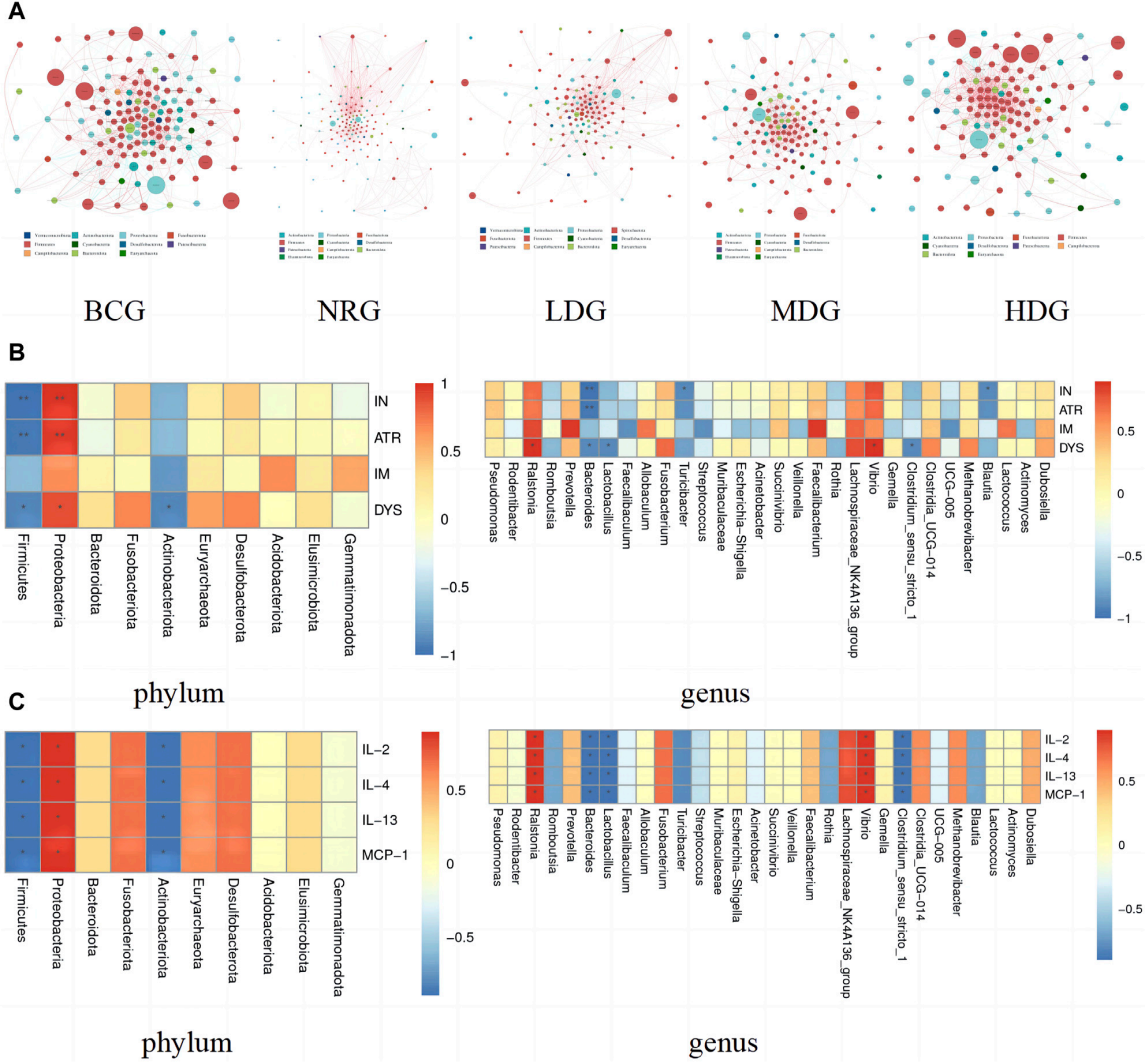
**(A)** Network of gastric mucosal microflora of rats in each group. **(B)** Pearson analysis of the pathology of gastric mucosa and gastric mucosal microflora (phylum and genus levels) Note: * indicates *p* < 0.05; ** indicates *p* < 0.01 **(C)** Pearson analysis of inflammatory factors and gastric mucosal microflora (phylum and genus levels) Note: * indicates *p* < 0.05; ** indicates *p* < 0.01.

The network of gastric mucosal microflora of rats in each group was calculated and drawn by Spearman correlation analysis. The dominant and closely interacting microflora in each group can be displayed intuitively. Refer to Figure 4A for details, in which the size of nodes represents the relative abundance, the red line between nodes indicates a positive correlation, and the blue line indicates a negative correlation. It can be found that the network density of the microflora with a positive correlation in the BCG is significantly higher than that with a negative correlation. On the contrary, the network density of the microflora with a negative correlation in the NRG is prominently higher than that with a positive correlation. While the ratio of the microflora with positive and negative correlations in the LDG, MDG, and HDG is between them. It is suggested that with the intervention of Weizhuan’an prescription, the gastric mucosal microflora has been remarkably regulated, which can play a better cooperative role and help restore the stability of gastrointestinal microecology.

#### 4.9.2 Correlation analysis between gastric mucosal pathology and gastric mucosal microflora

The correlation between the pathological indexes of the gastric mucosa and the dominant microflora in phylum (top 10) and genus (top 30) levels was calculated by Spearman’s correlation analysis. The Spearman correlation coefficient values were calculated by the corr.test function; then, the pheatmap function was used for visualization after testing the significance of the difference. Figure 4B shows that there is a correlation between the pathological changes of gastric mucosa and gastric mucosal microflora. Specifically, *Proteobacteria* was positively correlated with inflammation (IN), atrophy (ATR), and dysplasia (DYS) (*p* < 0.05, *p* < 0.01). *Ralstonia* and *Vibrio* were positively correlated with DYS (*p* < 0.05). *Prevotella* and *Faecalibacterium* were positively correlated with intestinal metaplasia (IM), while there was no statistical difference between the above groups (*p* > 0.05). *Firmicutes* and *Bacteroides* were negatively correlated with IN, ATR, and DYS (*p* < 0.05, *p* < 0.01). *Actinobacteriota* and *Lactobacillus* were negatively correlated with DYS (*p* < 0.05). *Turicibacter*, *Blautia*, and *Clostridium_sensu_stricto_1* were negatively correlated with IN (*p* < 0.05). In addition, it can also be found that the differential microflora has a higher correlation with IN, ATR, and DYS and a lower correlation with IM.

#### 4.9.3 Correlation analysis between gastric mucosal microflora and inflammatory factors

The results of Spearman correlation index analysis on gastric mucosal microflora and inflammatory factors indicate that there is a high correlation between them, see Figure 4C. Specifically, *Proteobacteria*, *Ralstonia*, and *Vibrio* were positively correlated with IL-2, IL-4, IL-13, and MCP-1 (*p* < 0.05). *Firmicutes*, *Actinobacteriota*, *Bacteroides*, *Lactobacillus*, and *Clostridium_sensu_stricto_1* were negatively correlated with IL-2, IL-4, IL-13, and MCP-1 (*p* < 0.05).

### 4.10 Functional abundance prediction

#### 4.10.1 Functional abundance prediction of gastric mucosal microflora in rats

**FIGURE 5:**
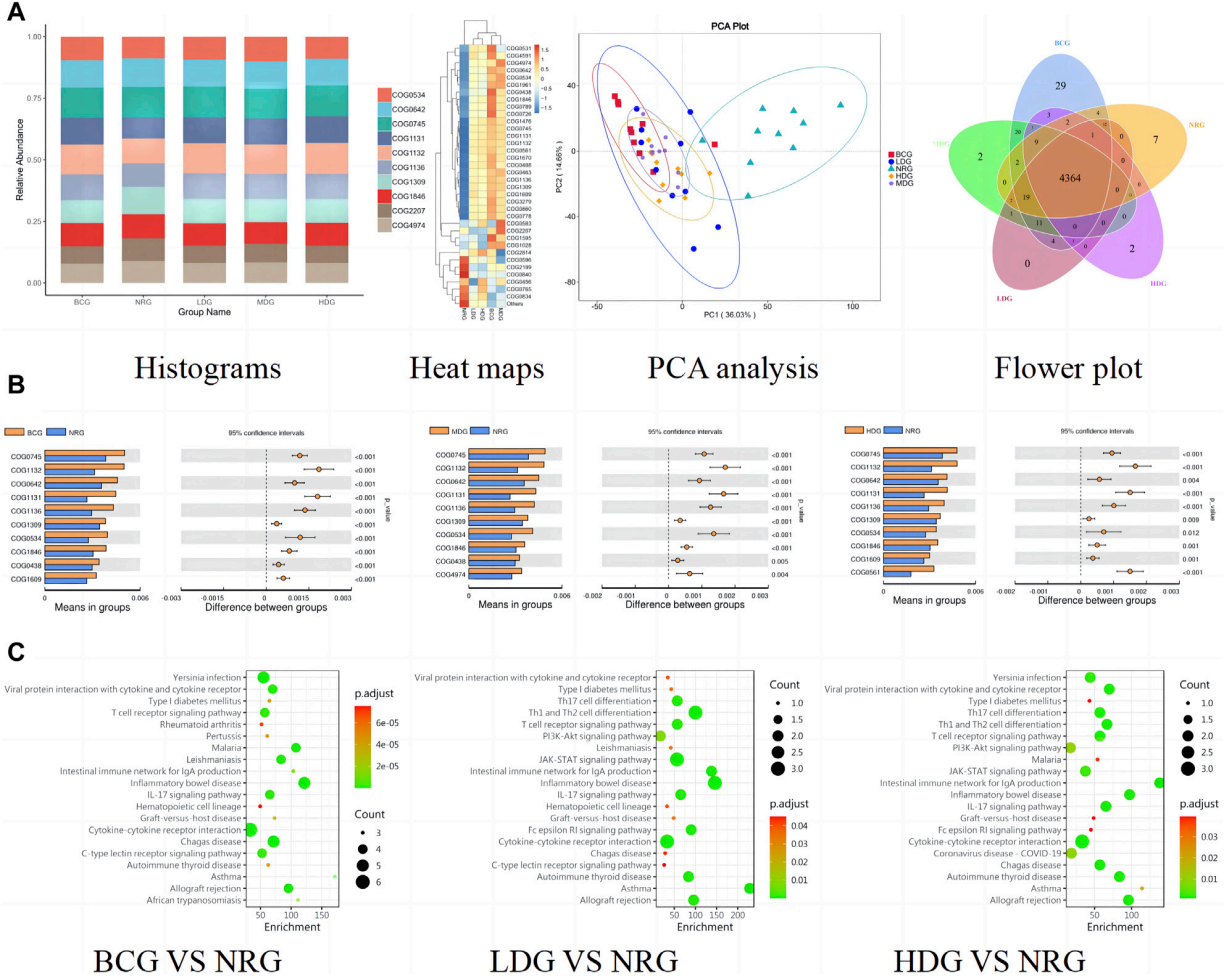
**(A)** Histograms, heat maps, PCA analysis, and flower plot of COG of rats in each group. **(B)** Bar charts of COG functional enrichment analysis of gastric mucosal microflora of rats in each group. **(C)** KEGG function enrichment analysis of gastric mucosal inflammatory factors of rats in each group.

The functional abundance prediction of gastric mucosal microflora was performed by PICRUSt2 based on the 16S rDNA sequencing data and the COG. First, functional abundance annotation and relative abundance clustering were conducted for the preliminary assessment of common and unique functional information between groups. The histograms (top 10), heat maps (top 35), PCA, and flower plot can be referred to Figure 5A. Then, the functional difference in gastric mucosal microflora in each group was analyzed by the *t* test. The results showed that compared with the NRG, the BCG, MDG, and HDG had significant differences (*p* < 0.05, *p* < 0.01). Especially, there were 2,123 different functions in the BCG, 2,198 in the MDG, and 2,160 in the HDG. See Figure 5B for the differential function with relatively high enrichment (top 10). It can be found that compared with the NRG, the DNA-binding transcriptional regulators such as OmpR family, AcrR family, and MarR family, ABC-type multidrug transport system and lipoprotein export system, and the activity of related enzymes were upregulated substantially in the BCG, MDG, and HDG. The related gastric mucosal microflora includes *Lactobacillus*, *Pseudomonas*, and *Faecalibacterium*. Refer to Table 5 for details.

**TABLE 5:** COG hierarchy and function description with related gastric mucosal microflora.

Hierarchy	Function description	Gastric mucosal microflora
COG0745	DNA-binding response regulator, OmpR family, contains REC and winged-helix (wHTH) domain	*Firmicutes*, *Romboutsia*, *Lactobacillus*, *Pseudomonas*, *Ralstonia*, *Vibrio*, *Prevotella*, *Faecalibacterium*, *Bacteroides*, *Blautia*, *Clostridium_sensu_stricto_1*, etc.
COG1132	ABC-type multidrug transport system, ATPase, and permease component	
COG0642	Signal transduction histidine kinase	
COG1131	ABC-type multidrug transport system, ATPase component	
COG1136	ABC-type lipoprotein export system, ATPase component	
COG1309	DNA-binding transcriptional regulator, AcrR family	
COG0534	Na^+^-driven multidrug efflux pump	
COG1846	DNA-binding transcriptional regulator, MarR family	
COG0438	Glycosyltransferase involved in cell wall biosynthesis	
COG1609	DNA-binding transcriptional regulator, LacI/PurR family	
COG4974	Site-specific recombinase XerD	
COG0561	Hydroxymethylpyrimidine pyrophosphatase and other HAD family phosphatases	

#### 4.10.2 Functional abundance prediction of gastric mucosal inflammatory factors in rats

The functional abundance prediction of differential inflammatory factors was performed by the KEGG enrichment analysis, which can screen out the biological regulatory pathways with significant differences between groups. It adopts the Fisher exact test, and the data package is clusterProfiler from R/Bioconductor. The criterion is that the count of genes of the pathway is ≥5, *p* < 0.05. The details are shown in Figure 5C (top 20). The results suggest that compared with the NRG, the signaling pathways significantly changed in the BCG, LDG, and HDG (*p* < 0.05, *p* < 0.01), such as viral protein interaction with cytokine and cytokine receptor, T cell receptor signaling pathway, intestinal immune network for lgA production, inflammatory bowel disease, and IL-17 signaling pathway. The related inflammatory factors include IL-1β, IL-2, IL-4, IL-13, TNF-α, IL-10, and MCP-1.

## 5 Discussion

GC is a serious threat to global human health. Fortunately, the PLGC is the effective intervention period for preventing GC. Several research studies have reported that TCM has many advantages in treating PLGC, such as individualization, good efficacy, economy, and non-invasiveness. As the empirical formula of our research group, Weizhuan’an prescription has been applied in the clinic for many years and has received satisfactory therapeutic feedback. We strictly standardize the basic experiments of botanical drugs and strive to combine modern medical research methods with TCM. In this experiment, 16S rDNA amplicon sequencing, cytokine antibody microarray, and Western blotting were applied to analyze the gastric mucosal microflora and inflammatory factors of rats to explore the biological mechanisms of Weizhuan’an prescription.

The results suggest that the pathological manifestations of gastric mucosa, the gastric mucosal microflora, and inflammatory factors in rats with PLGC have changed greatly compared with those of the BCG. With the intervention of Weizhuan’an prescription, the pathological improvement of the gastric mucosa was remarkable, especially in the MDG and HDG. The results of 16S rDNA amplicon sequencing showed that there were significant differences in the BCG, MDG, and HDG compared with the abundance and diversity of gastric mucosal microflora in the NRG. Specifically, at the phylum level, the abundance of *Firmicutes* increased significantly in the BCG (*p* < 0.01), and that of *Proteobacteria* decreased significantly in the BCG and MDG (*p* < 0.01). At the genus level, the abundance of *Romboutsia*, *Lactobacillus*, and *Turicibacter* increased in the BCG, and that of *Pseudomonas* decreased significantly in the BCG and MDG (*p* < 0.01). The abundance of *Lactobacillus* and *Veillonella* increased significantly in the HDG (*p* < 0.01). The results of cytokine antibody microarray and Western blotting indicated that compared with the NRG, the contents of IL-2, IL-4, IL-13, and MCP-1 were significantly decreased in the BCG, LDG, MDG, and HDG (*p* < 0.05).

*Firmicutes*, *Bacteroidetes*, *Proteobacteria*, and *Actinobacteria* are symbiotic bacteria in healthy intestines, which may turn into pathogenic bacteria in the unbalanced internal environment (Liu et al., 2022). *Proteobacteria* with high content can promote gastrointestinal inflammation by producing stimulating flagellin and lipopolysaccharide (Shin et al., 2015; Guo et al., 2022). This study also suggests that *Firmicutes* and *Bacteroides* can reduce inflammation, atrophy, and dysplasia of the gastric mucosa, while *Proteobacteria* may aggravate such lesions. *Pseudomonas* is a Gram-negative bacterium with a wide variety and distribution (Zha et al., 2015), including a variety of pathogens such as *Pseudomonas aeruginosa*, which is common in mucosal membrane damage and infection (Zhang XY. et al., 2021). This study found that *Pseudomonas* may promote gastric mucosa atrophy. *Romboutsia* is generally regarded as a probiotic, which can resist inflammation and protect mucosal barrier (Wu et al., 2021). *Turicibacter* can participate in metabolism and resist fatigue (Kemis et al., 2019; Chung et al., 2021). This study found that it can alleviate gastric mucosal inflammation. *Lactobacillus* is widely distributed in the digestive tract (Hindieh et al., 2022). As one of the most widely used probiotics in the clinic, *Lactobacillus* can reduce infection and inflammation, strengthen immunity, and maintain gastrointestinal health (Garcia et al., 2022; Hu et al., 2022). *Lactobacillus* can also inhibit the proliferation of cancer cells by producing anticancer metabolites and inducing apoptosis of cancer cells (Yue et al., 2021). This study found that *Lactobacillus* can alleviate gastric mucosal dysplasia. *Veillonella* is a kind of Gram-negative anerobic *micrococcus*, which can play an anti-inflammatory role and enhance the body’s immunity (Xiao et al., 2022; Daniel et al., 2022). In addition, the study found that the related microflora can significantly upregulate the activities of DNA-binding transcriptional regulators, ABC-type multidrug transport system, related enzymes, etc.; promote the synthesis of gastric epithelial cells and the absorption and utilization of effective drug metabolites; and help repair the damaged gastric mucosa to treat PLGC and prevent GC.

In recent years, with the progress of immunotherapy and the exploration of new targets in GC, significant breakthroughs have been made in the immunotherapy of GC. The molecular markers for the diagnosis and prognosis of GC include m1A, microRNAs, lncRNAs, and JMJD3 (Xu et al., 2019; Zhao et al., 2019; Chen Y. et al., 2021; Zhang HY. et al., 2021). It is also worth looking forward to whether immunotherapy can play a role in the treatment of PLGC. Moreover, it is suggested that *Proteobacteria* can increase the relative contents of IL-2, IL-4, IL-13, and MCP-1, while *Firmicutes*, *Bacteroides*, and *Lactobacillus* can decrease the contents of IL-2, IL-4, IL-13, and MCP-1 significantly. There is a close relationship between gastric mucosal inflammation and the generation of PLGC. Studies have shown that many botanical drug metabolites inhibit inflammatory reactions effectively (Chen QL. et al., 2021). Interleukin-2 (IL-2) is a pleiotropic cytokine, which has dual regulatory effects on immune activation and immune tolerance (Yui et al., 2004; Hershko et al., 2011). IL-2 can activate cytotoxic T cells and NK cells to participate in the inflammatory reaction (Sockolosky et al., 2018). Furthermore, IL-2 can promote the proliferation and differentiation of Treg cells (Sharma et al., 2020), and Treg cells can inhibit the activity of cytotoxic T cells to induce immune escape, tumor recurrence, or metastasis (Najafi et al., 2019). The sustained high levels of IL-2 can also induce toxicity (Dutcher et al., 2014). Interleukin-4 (IL-4) is also a “double-edged sword” as a kind of pleiotropic cytokine. On one hand, it can play an anti-tumor role, such as promote the differentiation of B cells (Zubiaga et al., 1992) and stimulate the production of CD_8_^+^ T cells (Schultze et al., 1997). On the other hand, studies (Suzuki et al., 2015; May and Fung, 2015) confirmed that the level of IL-4 in cancer patients is higher, and there is a high level of IL-4R receptor expression on the surface of tumor cells. Tumor cells can also produce IL-4 (Todaro et al., 2008), which can activate tumor-associated macrophages (TAMs) and myeloid-derived suppressor cells (MDSCs), thus enhancing the invasion of tumors (Suzuki et al., 2015). Interleukin-13 (IL-13) is a pleiotropic Th2 cytokine (Heeb et al., 2020; Moran and Pavord, 2020). It can activate eosinophils, enhance the inflammatory response, and damage the mucosal barrier (Tsou et al., 2015; Sato et al., 2017). In the inflammatory microenvironment, IL-13 can also cooperate with IL-4 to promote the development of tumors through autocrine and paracrine paths (Surana et al., 2014; Bankaitis and Fingleton, 2015). Monocyte chemotactic protein-1 (MCP-1), also known as CCL2, is an important pro-inflammatory factor (Qian et al., 2011). It can activate monocytes, macrophages, and T cells and actively chemotactic immune cells to participate in inflammatory activities. In the inflammatory microenvironment, MCP-1 can enhance the activity of tumor cells and promote the generation of tumor microvessels and lymphatic vessels (Qian et al., 2009; Tang and Tsai, 2012). MCP-1 is also a potent chemokine of TAMs, resulting in tumor immune escape (Roblek et al., 2016). This study also suggests that inflammatory factors IL-2, IL-4, IL-13, and MCP-1 are related to the change of multiple signaling pathways, such as viral protein interaction with cytokine and cytokine receptors, T cell receptor signaling pathway, intestinal immune network for lgA production, inflammatory bowel disease, and IL-17 signaling pathway.

However, the interaction mechanism between botanical drug, microflora, and PLGC is complex, and the number of related genes, proteins, and signal pathways is huge. Further exploration will be carried out with the help of related technologies in order to further clarify the molecular network mechanism of the effective metabolites of botanical drug, key microflora, and PLGC and provide more references for clinical practice.

## 6 Conclusion

Weizhuan’an prescription can significantly improve the gastric mucosa pathology of rats with PLGC by regulating the gastric mucosal microflora and inflammatory factors; increasing the species and abundance of probiotics; reducing the content of pathogenic bacteria and IL-2, IL-4, IL-13, and MCP-1; and regulating multiple signal pathways.

## Data Availability

The data of this paper is stored in SRA database. The accession number is PRJNA1144098, available at: https://www.ncbi.nlm.nih.gov/sra/PRJNA1144098.
